# Application of Novel Psychoactive Substances: Chemsex and HIV/AIDS Policies Among Men Who Have Sex With Men in Hong Kong

**DOI:** 10.3389/fpsyt.2021.680252

**Published:** 2021-07-14

**Authors:** Alex Siu Wing Chan, Patrick Ming Kuen Tang

**Affiliations:** ^1^Department of Applied Social Sciences, The Hong‘Kong Polytechnic University, Hong Kong, China; ^2^Department of Anatomical and Cellular Pathology, State Key Laboratory of Translational Oncology, The Chinese University of Hong Kong, Hong Kong, China

**Keywords:** LGBTQ, HIV/AIDS infectious diseases, public health policies and intersectoral collaboration, chemsex, MSM

Chemsex is identified as the use of several drugs shortly before or during sexual intercourse in order to stimulate, extend, and/or enhance sexual encounters, in particular in certain groups of men who have sex with men (MSM) (1). It is observed that the frequency of chemsex among one research group, MSM attending Dutch STI centers in Limburg, reached 35%. Chemsex is already implicated in the development of sexually transmitted infections (STIs) as well as a higher likelihood of addiction, anxiety, and depression (2, 3). Chemsex has been recognized as a worldwide phenomenon, and there has been a rise in overall medical and academic concerns over chemsex, whereby substance use is linked to boosting sexual satisfaction, typical in a group setting, especially among homosexuals. It is reassuring to learn that lesbian, gay, bisexual, transgender, and queer (LGBTQ) individuals have transformed into an increasingly committed, valued, and visible group in society (4). However, the issue of chemsex continues to be one that must be addressed expeditiously. A strategy based on ultra-performance liquid chromatography–tandem mass spectrometry was created to measure the most frequently abused substances, synthetic cannabinoids, synthetic cathinones, and GHB, in the nails of people suspected of taking such drugs in music and intercourse contexts (5, 6). Prescription drug abuse and its associated hazards, including co-ingestion with recreational substances, have lately come to prominence as a global general well-being issue. These could have a number of healthcare and societal effects, necessitating strong community healthcare strategies to combat the practice, along with ongoing education and training for health professionals to enhance awareness and harm minimization (7). As a result, casual encounters are being rendered extremely convenient *via* mobile devices. These are a shared means of relief that improves the welfare of homosexuals in an environment that discriminates against them. The substances seen in such events are primarily γ-hydroxybutyrate (GHB) and γ-butyrolactone (GBL), or G, crystallized methamphetamine, and mephedrone (8). Kong and Laidler (9) note that this chemsex trend will ultimately become a major concern, harming homosexual men who employ chem to fulfill their sexual needs as well as causing HIV/AIDS.

Lately, problems associated with HIV/AIDS and substance not only concern Hong Kong, but everywhere in the globe. Kong and Laidler (9) tracked the implementation of neo-liberal narratives about local HIV/AIDS culture and drug laws. The study indicates that this emerging problem is the product of two conflicting policy directions, resulting in a crucial divide in medical care. Therefore, the research effectively explores the link between HIV/AIDS and drug regulations in a unique social sense, exposing patient care inequalities and enhancing our knowledge of the development of capitalist topics in healthcare services outside of the West.

Every government on the planet is concerned about drug policies. This research reviews Hong Kong's drug legislation, which is important for the elimination of HIV/AIDS risks. Kong and Laidler (9) present community health perspectives to explain the minority sexual inhibition and reinforcement of the efficacy of specific medications (i.e., crystal meth, poppers, ecstasy, ketamine, GBH, and mephedrone), with a special emphasis being placed on the application of novel psychoactive substances (NPS), which are both strong and immensely satisfying and linked to MSM chemsex as well as their ability to result in more HIV and sexual diseases, which discusses the application of NPS and how they are positioned within a “postmodern syndrome” (10). The study also mentions many locations all over the U.S., Australia, the U.K., Africa, and Western Europe to demonstrate that chemsex is a significant problem that has a huge influence on sexual welfare today. Earlier findings indicated that MSM who participate in chemsex display a plethora of different characteristics about their sexual activity and sexual well-being, such as a greater risk for HIV infection than MSM who do not participate in chemsex (11, 12), along with an increased proportion of STIs and a greater likelihood of hepatitis C transmission (13). This is already a concerning trend, but what makes it worse is that chemsex is closely correlated with sex in public. Kong and Laidler (9) stress that chemsex is often linked to public sex involvement, having sex with many individuals, and more risky sex acts, such as anal sex without the use of condoms between HIV-negative men and partners who are infected with HIV or whose HIV status is unclear. It is worth noting that interaction with LGBT people has been identified as beneficial to the general well-being of such individuals (14). The issue now appears to increase the risk of broad substance use, particularly in terms of sex among MSM. Several MSM groups may have a different societal standard for drug use. For example, recent findings suggest that drug use is prevalent among their acquaintances. Inevitably, unrestrained drug use among MSM puts their health at risk, which is detrimental to the development of a sustainable homosexual community.

In view of this, the article explores the question of how to work together to develop a solid homosexual culture. It specifically explains the role of human services, for example, improving policy frameworks in HIV-related healthcare facilities, to allow workers to recognize MSM who use substances as well as offering related resources and appropriate recommendations. Meanwhile, consideration must be given to the implementation and enhancement of HIV control and monitoring programs in drug recovery and medical care under the Hospital Authority and non-governmental organizations. Every group should strive for enhanced coordination between HIV programs and drug-related programs. Unrestricted exposure to HIV-related facts and resources is of extreme significance. Citizens must also be adequately equipped with knowledge to cultivate a discrimination-free atmosphere. The utilization of facilities associated with HIV ought to be supervised. Some members of the MSM expressed their serious reservations about stereotypes and sexism, while some actively support the legal rights of homosexual people. In any case, a systematic approach to advancing homosexual rights is required if meaningful progress is to be made. This scientific proof recommendation guides public health initiatives, service delivery, and institutional arrangements concerning NPS supervision and oversight. Nevertheless, in the absence of dependable, precise, and proper records that are accurately compiled, scientifically analyzed, and distributed in a timely manner, a picture of the concepts as to what fatalities can be attributed to NPS, their attributes, and essence will remain unattainable, restricting how much can be done to minimize them (15). GBL is presently an increasing health risk, owing to the fact that it is less expensive and simpler to get than GHB. GBL laws and regulations must be improved or implemented to restrict its spread, the future medical harm to people, and the misuse accountability and addiction issues that cannot be ignored (5). Thus, credible proof of the effects of related legal concerns on the MSM HIV crisis must be retained in sight.

To conclude, individuals who have excellent mental health are more easygoing and live a more energetic and pleasant life (16), whereas homosexual men have a much higher risk of harm to their mental health with chemsex behavior (17). In addition, while homosexual men who indulge in chem-fun should be punished by neoliberal logic, these individuals are not violating any substances or HIV/AIDS legislation considering that consuming sex drugs puts them beyond these policy boundaries. Given that specific services to address their unique needs are not available, and drug and medical professionals are not well-prepared to offer both HIV/AIDS and drug use education and training, this is a tricky issue to address. Fortunately, by revealing the disparity between HIV/AIDS and drug laws in Hong Kong, this study strengthens our comprehension of the development of a neo-liberal topic across prohibitionist and damage reduction frameworks outside of the West. Nevertheless, much work remains to be done in the short term, and until a feasible and comprehensive solution is developed, unsafe sexual activities, binge drinking and drug misuse, and poor HIV screening and intervention rates remain main issues for the MSM population.

## Author Contributions

AC was mainly writing this manuscript. PT gave his professional clinical advice and suggestion in the discussion part. All authors contributed to the article and approved the submitted version.

## Conflict of Interest

The authors declare that the research was conducted in the absence of any commercial or financial relationships that could be construed as a potential conflict of interest.
